# Supplementary material for: End Sequence Analysis Toolkit (ESAT) expands the extractable information from single-cell RNA-seq data
Source: Genome Res. 2016 Oct;26(10):1397–410. doi: 10.1101/gr.207902.116 (PMC5052061; doi:10.1101/gr.207902.116)
Supplement: Supplemental Material [file supp_gr.207902.116_Supp_Materials.tar.gz › ESAT-esat_v0.1/doc/html/classbroad_1_1pda_1_1seq_1_1segmentation_1_1_gene_score.html]

ESAT: broad.pda.seq.segmentation.GeneScore Class Reference


ESAT


- Main Page
- Related Pages
- Packages
- Classes
- Files

- Class List
- Class Index
- Class Hierarchy
- Class Members

All Classes Namespaces Files Functions Variables Enumerator Pages

- broad
- pda
- seq
- segmentation
- GeneScore

Public Member Functions |
List of all members

broad.pda.seq.segmentation.GeneScore Class Reference

Inheritance diagram for broad.pda.seq.segmentation.GeneScore:

[legend]

Collaboration diagram for broad.pda.seq.segmentation.GeneScore:

[legend]

|  |  |
| --- | --- |
| Public Member Functions | |
|  | GeneScore (Gene gene, double[] scores) |
|  | |
|  | GeneScore (Gene gene) |
|  | |
| void | setEnrichment (double enrichment) |
|  | |
| double | getEnrichment () |
|  | |
| void | setNumberOfReads (double numberOfReads) |
|  | |
| double | getNumberOfReads () |
|  | |
| void | setAvergeNumberOfReads (double avergeNumberOfReads) |
|  | |
| double | getAvergeNumberOfReads () |
|  | |
| void | setRPKM (double rPKM) |
|  | |
| double | getRPKM () |
|  | |
| void | setLocalLambda (double localLambda) |
|  | |
| double | getLocalLambda () |
|  | |
| void | setGeneLength (double geneLength) |
|  | |
| double | getGeneLength () |
|  | |
| void | setNominalPValue (double nominalPValue) |
|  | |
| double | getNominalPValue () |
|  | |
| void | setScanPvalue (double scanPvalue) |
|  | |
| double | getScanPvalue () |
|  | |
| void | setFullyContainedNumberOfReads (double fullyContainedNumberOfReads) |
|  | |
| double | getFullyContainedNumberOfReads () |
|  | |

## Constructor & Destructor Documentation

|  |  |  |  |
| --- | --- | --- | --- |
| broad.pda.seq.segmentation.GeneScore.GeneScore | ( | Gene | *gene*, |
|  |  | double[] | *scores* |
|  | ) |  |  |

A class to represent the standard scores assigned to a window or Gene in the ContinuousDataAlignmentModel

|  |  |  |  |  |  |
| --- | --- | --- | --- | --- | --- |
| broad.pda.seq.segmentation.GeneScore.GeneScore | ( | Gene | *gene* | ) |  |

## Member Function Documentation

|  |  |  |  |  |
| --- | --- | --- | --- | --- |
| double broad.pda.seq.segmentation.GeneScore.getAvergeNumberOfReads | ( |  | ) |  |

|  |  |  |  |  |
| --- | --- | --- | --- | --- |
| double broad.pda.seq.segmentation.GeneScore.getEnrichment | ( |  | ) |  |

|  |  |  |  |  |
| --- | --- | --- | --- | --- |
| double broad.pda.seq.segmentation.GeneScore.getFullyContainedNumberOfReads | ( |  | ) |  |

|  |  |  |  |  |
| --- | --- | --- | --- | --- |
| double broad.pda.seq.segmentation.GeneScore.getGeneLength | ( |  | ) |  |

|  |  |  |  |  |
| --- | --- | --- | --- | --- |
| double broad.pda.seq.segmentation.GeneScore.getLocalLambda | ( |  | ) |  |

|  |  |  |  |  |
| --- | --- | --- | --- | --- |
| double broad.pda.seq.segmentation.GeneScore.getNominalPValue | ( |  | ) |  |

|  |  |  |  |  |
| --- | --- | --- | --- | --- |
| double broad.pda.seq.segmentation.GeneScore.getNumberOfReads | ( |  | ) |  |

|  |  |  |  |  |
| --- | --- | --- | --- | --- |
| double broad.pda.seq.segmentation.GeneScore.getRPKM | ( |  | ) |  |

|  |  |  |  |  |
| --- | --- | --- | --- | --- |
| double broad.pda.seq.segmentation.GeneScore.getScanPvalue | ( |  | ) |  |

|  |  |  |  |  |  |
| --- | --- | --- | --- | --- | --- |
| void broad.pda.seq.segmentation.GeneScore.setAvergeNumberOfReads | ( | double | *avergeNumberOfReads* | ) |  |

|  |  |  |  |  |  |
| --- | --- | --- | --- | --- | --- |
| void broad.pda.seq.segmentation.GeneScore.setEnrichment | ( | double | *enrichment* | ) |  |

|  |  |  |  |  |  |
| --- | --- | --- | --- | --- | --- |
| void broad.pda.seq.segmentation.GeneScore.setFullyContainedNumberOfReads | ( | double | *fullyContainedNumberOfReads* | ) |  |

|  |  |  |  |  |  |
| --- | --- | --- | --- | --- | --- |
| void broad.pda.seq.segmentation.GeneScore.setGeneLength | ( | double | *geneLength* | ) |  |

|  |  |  |  |  |  |
| --- | --- | --- | --- | --- | --- |
| void broad.pda.seq.segmentation.GeneScore.setLocalLambda | ( | double | *localLambda* | ) |  |

|  |  |  |  |  |  |
| --- | --- | --- | --- | --- | --- |
| void broad.pda.seq.segmentation.GeneScore.setNominalPValue | ( | double | *nominalPValue* | ) |  |

|  |  |  |  |  |  |
| --- | --- | --- | --- | --- | --- |
| void broad.pda.seq.segmentation.GeneScore.setNumberOfReads | ( | double | *numberOfReads* | ) |  |

|  |  |  |  |  |  |
| --- | --- | --- | --- | --- | --- |
| void broad.pda.seq.segmentation.GeneScore.setRPKM | ( | double | *rPKM* | ) |  |

|  |  |  |  |  |  |
| --- | --- | --- | --- | --- | --- |
| void broad.pda.seq.segmentation.GeneScore.setScanPvalue | ( | double | *scanPvalue* | ) |  |

---

The documentation for this class was generated from the following file:

- java/broad/pda/seq/segmentation/GeneScore.java


---

Generated on Fri Aug 15 2014 15:11:05 for ESAT by  

 1.8.7
